# A Series of Asymmetrical Phthalocyanines: Synthesis and Near Infrared Properties

**DOI:** 10.3390/molecules18044628

**Published:** 2013-04-19

**Authors:** Guoqing Huang, Jianxi Li, Fangdi Cong, Chao Li, Xixi Chu, Yanyan Meng, Guotong Du, Xiguang Du

**Affiliations:** 1 State Key Laboratory on Integrated Optoelectronics, College of Electronic Science and Engineering, Jilin University, Changchun 130012, China; 2 Department of Chemistry, Northeast Normal University, Changchun 130024, China

**Keywords:** organic green dyes, syethesis, NIR-absorption, NIR luminescence, 1-D hexrod nanotubes

## Abstract

We report here the preparation of asymmetrical phthalocyanine dimers **1a**–**3a**, which are endowed with novel charge transfer bands at 1,151–1,154 nm and strong NIR luminescences at 840–860 nm and 1,600–1,650 nm. Through H-bonding interaction, **1a**–**3a** are inclined to self-assemble into hexrod nanotubes at the interface of CHCl_3_ and CH_3_OH. Our results provide further insights into the interaction in molecular dimers, and suggest that **1a**–**3a** have potential application in magnets and supramolecular architectures.

## 1. Introduction

Considerable efforts have been made to develop various types of supramolecular architectures formed by self-organization between functional chromophores, because these supramolecular architectures have better photophysical and electronic properties as a result of excitonic interactions [1,2,3,4,5,6,7,8]. Phthalocyanines (Pcs), as a type of functional chromophores, have potential application in supermolecular assemblies, charge transfer properties, and magnets [9], such as NIR-absorbing Pc supermolecules with some unique electronic spectrum and electrochemical behaviors [10,11,12,13,14,15,16]. Among them, double-decker Pc dimers have been of specially interest in numerous contexts because they are inclined to generate intermolecular π-π interactions [17,18]. Especially those with new CT bands in the range of 1,100–1,280 nm (in oxidized state) have potential applications in emerging photoelectric technologies [19,20]. Some self-organization structures of Pcs were reported by other groups [21].

Of particular interest in a number of contexts are Pcs which self-assemble and self-organize into 1D nanostructures. Recently, Pcs that self-organize by H-bonding, electrostatic, labile metal-ligand bonds and flat π-π interaction into highly ordered supramolecular structures with controlled dimensions and size, have attracted great interest, such as Nolte and co-workers’ reports of a Pc with four crown ether moieties, which are self-organized into helical, micrometer-long fibers in chloroform solution [22]. Ultralong nanowires with new crystal structured are formed by CuPc [23]. Although scientists studying Pcs have reported some 1D nanostructures [24,25], 1D nano-tubular structures produced by self-assembly methods have seldom been reported.

Our previous research work on the design and synthesis of metallo-Pcs and Pc 1D tubular structures [26,27,28], helped us find and obtain A_3_B asymmetrical Ni (II) Pcs **1a**–**3a**, which are connected with three phenoxy groups and one benzyloxy group. It is found that **1a**–**3a** are self-assembled into hexo-nanotubes by H-bonding interactions between Pc molecules. The mechanism of formation of the hexotubular structures is proposed.

## 2. Results and Discussion

### 2.1. Synthesis

**Scheme 1:**
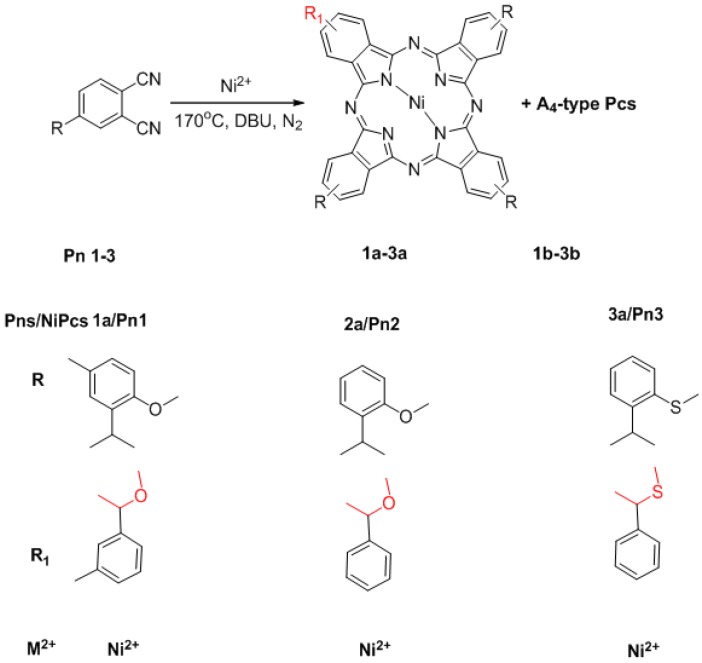
Synthesis of **1a**–**3a**.

The synthesis of **1a**–**3a** is summarized in Scheme 1. Only the cyclic tetramerization of single **Pn1-3** (Scheme S1 and Figure S1) in 1-octanol in the presence of 1,8-diaza-bicyclo[5.4.0] undec-7-ene (DBU) and at higher temperature of 170 °C (the traditional method use 140 °C), yields **1a**–**3a** and **1b**–**3b** (A_3_B-type Pcs and A_4_-type Pcs). 

*Reagents and Conditions*: 1-octanol (5 mL), DBU (2 mL), 170 °C, NiCl_2_·6H_2_O, 8 h.

The yield of **1a** is 15%, and the **2a** and **3a** is 13% and 8% respectively. This method overcomes many of the separation difficulties typically encountered in the traditional syntheses of asymmetrical Pcs [29] and only uses a single substituted phthalonitrile to synthesize asymmetrical Pcs (the traditional methods use two substituted phthalonitriles). A possible mechanism for the synthesis of **1a**–**3a** is provided in the Supporting Information (Scheme S2). Compounds **1a**–**3a** were characterized by UV-Vis-NIR (Figure S2), ^1^H-^13^C-NMR (Figure S3), high resolution MS and MALDA-TOF MS (Figure S4), IR (Figure S5). Characterization of **1a**, as an example of **1a**–**3a**, is discussed below. The data of **2a**–**3a** are provided in the Supporting Information.

### 2.2. High Resolution MS

**Figure 1:**
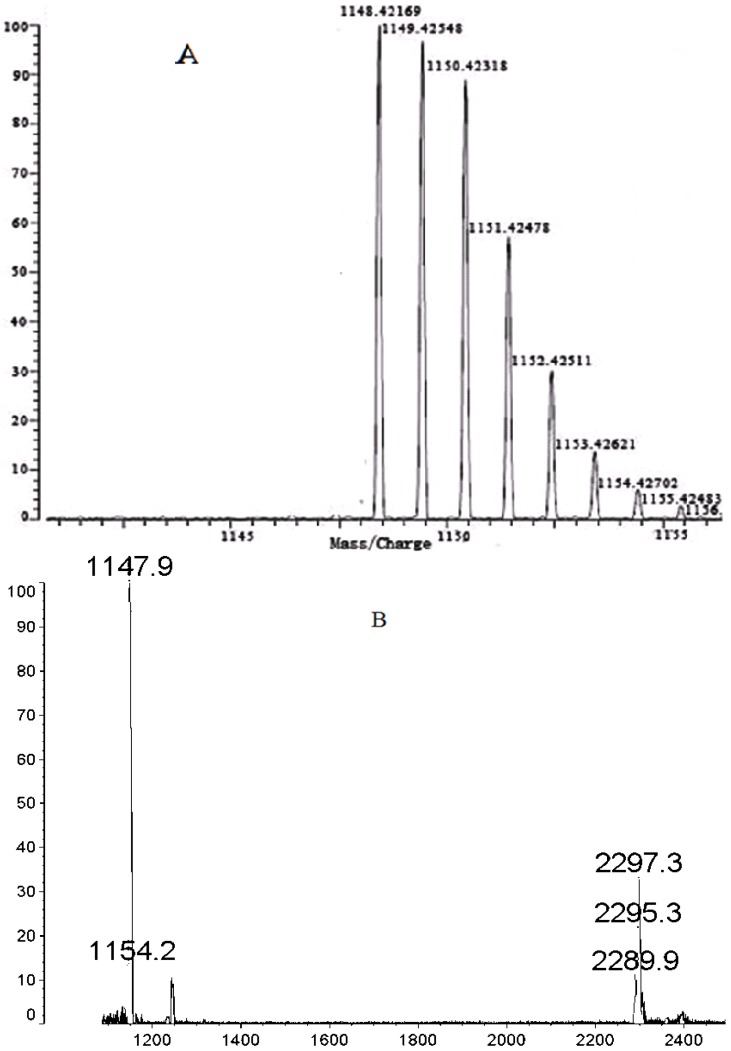
(**A**) Hi-Res MS spectrum of **1a**; (**B**) MALDI-TOF MS spectrum of **1a** dimer.

Figure 1A shows the clear *m/z* peaks ([M+H^+^] or M^+^) of **1a** obtained in high resolution MS. It is found that the molecular weight of **1a** is 1,148.42169, which is in line with the real molecular weight. The *m/z* peak at 2,297.3 provides direct evidence that **1a** tends to form a **1a** dimer (Figure 1B).

### 2.3. ^1^H-^13^C-NMR

**Figure 2:**
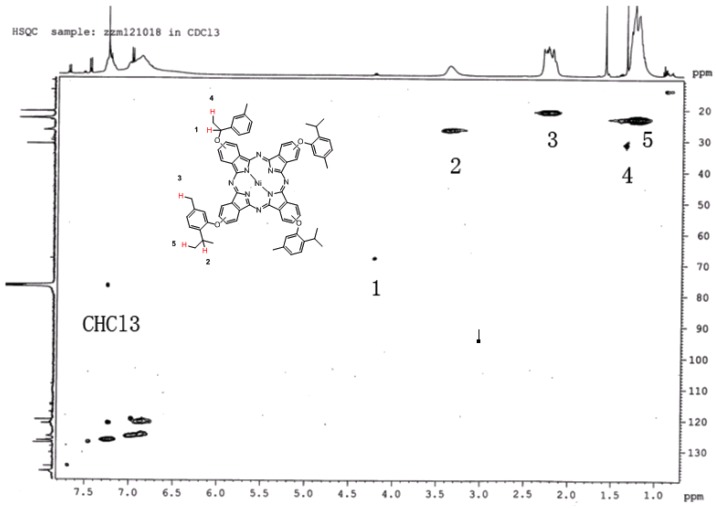
The ^1^H-^13^C-NMR of **1a**.

Because of the magnetism of **1a**, the peak area is influenced. The structure of **1a** is characterized by ^1^H-^13^C-NMR (in CDCl_3_) in Figure 2. We can see that label 1 is assigned to the C and H atoms in the benzyloxy group, label 2 is assigned to the C and H atoms in the isopropyl group, label 3 is the C and H atoms of the methyl group linked to the benzene ring, label 4 is the C and H atoms of the hypo-methyl group in the benzyloxy structure and the label 5 is the C and H atom of methyl group in isopropyl. The structure of **1a** was confirmed by ^1^H-^13^C-NMR and high resolution MS.

### 2.4. UV-Vis-NIR Spectroscopy

**Figure 3:**
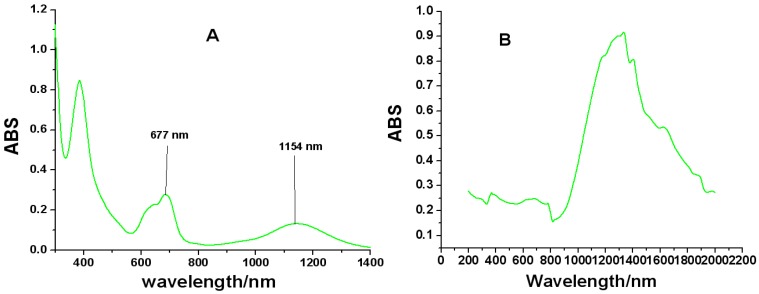
(**A**) UV-Vis/NIR spectrum of **1a** in CHCl_3_ (1.0 × 10^−4^ mol/dm^3^) and (**B**) Solid state UV-Vis/NIR Spectrum of **1a**.

**Figure 4:**
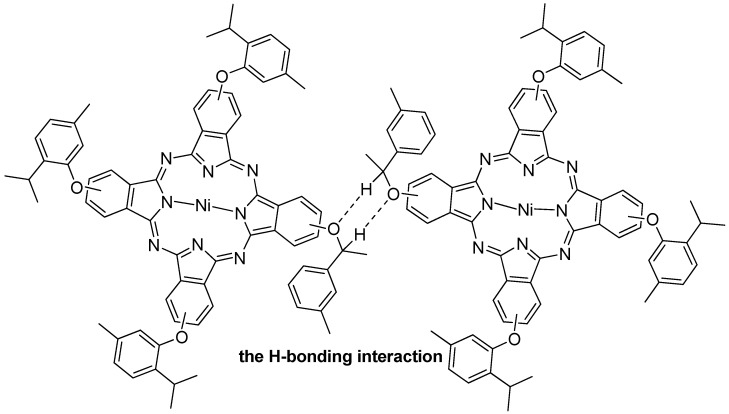
**1a** dimer in H-bonding interaction.

The same conclusion concerning the aggregation properties of the molecule were also obtained using UV-Vis-NIR spectroscopy (Figure 3). The B, Q and shoulder bands of **1a** in CHCl_3_ (1 × 10^−4^ mol/L) are observed around 385–390, 610 and 677 nm, respectively. It is noteworthy that the Q band of **1a** broadens considerably relative to the symmetrical one (Figure S6), but its molar extinction coefficient decreases dramatically from log *ε* 5.35 to log *ε* 2.89 (Figure 3A), which are just the characteristic absorptions of Pc dimers and oligomers [30]. Additionally, a broad intensity-strong band at around 1,154 nm is attributed to **1a** dimer, which is assignable to a CT consequence of electronic coupling due to strong H-bonding interaction in-between the **1a** dimer units (Figure 4). Compared with **2a**, the Q band and the NIR band of **3a** is red shifted from 1,148 nm to 1,230 nm, because the donating electron ability of phenylthiol group is better than that of a phenoxy group. The solid state UV-Vis-NIR spectrum of **1a** dimer shows that the molecule strongly NIR-absorbs light from 900 to 2,000 nm (Figure 3B), which hints that **1a** may exist in a face-to-tail *J*-type aggregates in the solid state and **1a** film might be improved as an available NIR material in optical communication field in the future.

### 2.5. NIR Luminescence

**Figure 5:**
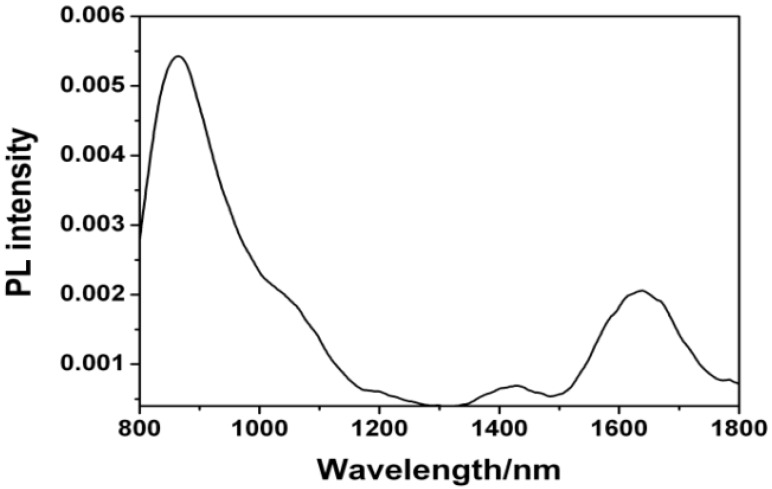
Emission spectrum of **1a** at room temperature.

To further confirm the CT capacity of dimer, we tested the NIR luminescence properties of **1a** by a 325 nm He-Cd laser as the excitation source and Ge detector (Figure 5). When **1a** is excited in KBr, it showed strong NIR luminescence at 840–860 nm and 1,600–1,650 nm, respectively. The NIR luminescence at 840–860 nm results from the fluorescent radiation transition (S_1_→S_0_) in the monomer, while the luminescence at 1,600–1,650 nm comes from the fluorescent radiation monomer-to-monomer transition. The results provide direct evidence of CT action in **1a** dimer.

### 2.6. 1-D Molecular Morphology and XRD

**Figure 6:**
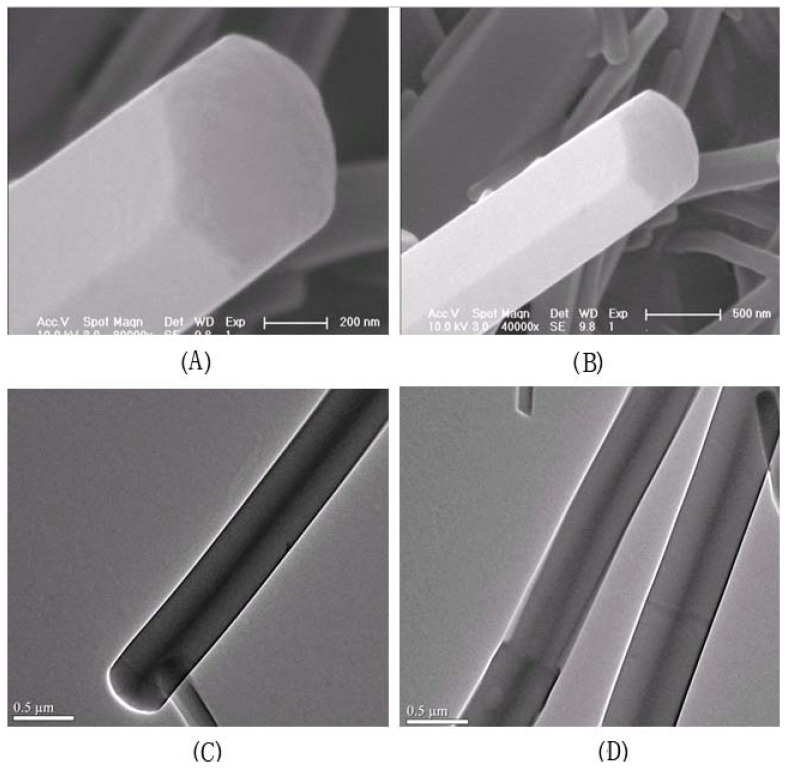
(**A**,**B**) SEM images of **1a** nanotubes and (**C**,**D**) HR-TEM micrographs of **1a** nanotubes.

Figure 6 shows 1-D molecular morphology of **1a**, which was tested by scanning electron microscope (SEM, Figure 6A,B) and high resolution transmission electron microscope (HR-TEM, Figure 6C,D). The SEM images show **1a** is self-assembled into hexrod nanotubes at the CHCl_3_ and CH_3_OH interface, and the nanotubes differ in length and thickness. The HR-TEM images show the hexrod-nanotubes are hollow, and the black lines in the HR-TEM images is the edges of nanotubes. These nanotubes indicate strong intermolecular aggregation in-between **1a** units. 

**Figure 7:**
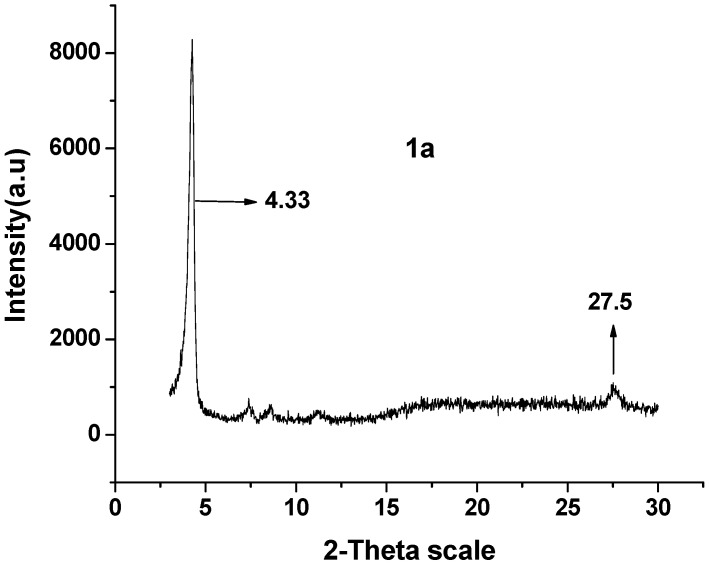
XRD data of **1a**.

The XRD spectrum of **1a** (Figure 7) shows two clear diffraction peaks at 4.33° and 27.5°, whose plane separations are approximately 21.45 Å (*d_1_*) and 3.098 Å (*d_2_*), respectively. The two peaks of the nanotubes shows that the **1a** molecules have been stacked in highly long-range ordered lamellar structure [31], *d_1_* and *d_2_* are the interlayer spacing of the nanotubes [32].

**Figure 8:**
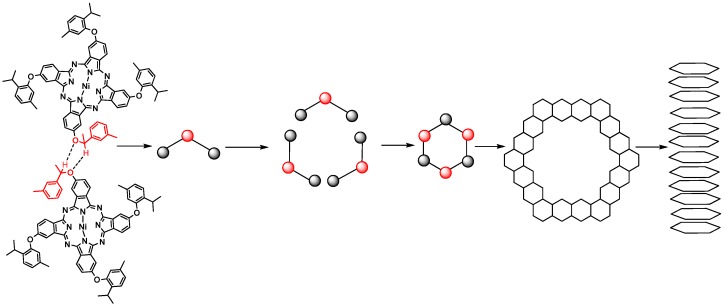
The possible formation of **1a** hexrod nanotubes.

Connecting the molecular morphology with the XRD data of **1a**, the possible formation of **1a** nanotubes can be described as shown in Figure 8: (1) the H-bonding interaction in-between **1a** is self-assembled into **1a** dimer. The angle of **1a** dimer is 120°, which is similar to the H-bonding interaction of H_2_O; (2) according to “proximity compatibility principle”, the **1a** dimer is self-assembled into a hexacyclic compound; (3) through *π-π* interaction in *J*-type aggregates [33], the hexacyclic compound forms nanotubes differing in length and thickness.

### 2.7. Magnetic Susceptibility

**Figure 9:**
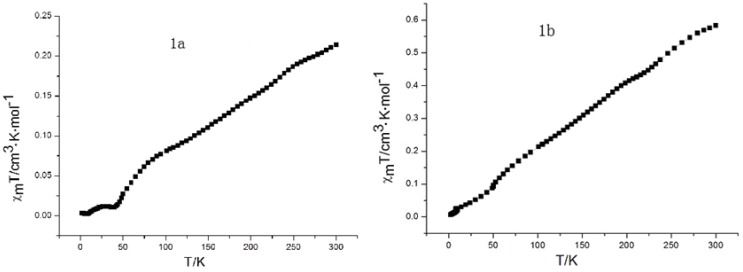
χm^T^ curves of magnetic measurements for **1a** and **1b**.

Magnetic susceptibility data of **1a** and **1b** were recorded using a Quantum Design SQUID MPMS XL-5 magnetometer. Variable temperature susceptibility measurements were carried out in the temperature range of 2–300 K at a magnetic field of 1,000 Oe on polycrystalline samples. Since Ni(II)Pc is diamagnetic due to the electronic configuration *(b_2g_)^2^(e_g_)^4^(a_1g_)^2^*, the paramagnetism of **1a** and **1b** should arise from the spin polarization of electrons on the aromatic macrocycle [34,35]. Both **1a** and **1b** with Ni(II) ion appear as planar molecules with no axial ligand, so their crystals are formed from various stacking columnar structures with different tilting angles and interplanar distances. As seen in Figure 9, upon continuously lowering temperature from 300 K to 2 K, the χm^T^ values of **1a** and **1b** smoothly decreases to a zero value; this is because the antiferromagnetic exchange interaction occurs between the radical aromatic macrocycle complexes in the solid state.

## 3. Experimental 

### 3.1. Experimental Materials and Equipment

Chloroform was distilled from CaH_2_ under nitrogen. DMSO was distilled from molecular sieves. The other reagents were purchased from commercial sources and used without further treatment. ^1^H-NMR and ^13^C-NMR spectra were recorded on a Varian 500 MHz instrument at 25 °C. Mass spectra were recorded on a Hi-Res MALDI and a LDI-1700 MALDI-TOF MS spectrometer. IR spectra (KBr) were recorded on a Magna-560 FT-IR spectrophotometer in the 400~4,000 cm^−1^ range. UV/VIS/NIR spectra were recorded on a Cary 500UV-VIS-NIR spectrophotometer. 

### 3.2. Synthesis of Substrates and Pc Derivatives

#### 3.2.1. General Procedure for the Synthesis of Substrates **Pn1**–**Pn3**

Lithium hydroxide hydrate (4.20 g, 0.10 mol) was interfused at room temperature into a stirred mixture of the appropriate phenol/thiophenol (2-isopropyl-5-methylphenol, 6.08 g, 0.04 mol for **Pn1**, 2-isopropylphenol, 5.44 g, 0.04 mol for **Pn-2** or 2-isopropylphenylthiol, 6.08 g, 0.04 mol for **Pn-3**) and 4-nitrophthalonitrile (6.92 g, 0.04 mol) in dimethylsulfoxide (80 mL) over a 2 h period. The mixture was stirred unceasingly and the reaction was monitored by thin-layer chromatography. After 24 h, the reactant was poured into NaCl solution (400 mL, 10%) and stirred till precipitate appeared. The product was collected by vacuum filtration and purified by column chromatography (silica gel/petroleum ether–ethyl ether 1:1) to afford **Pn-1-Pn-3**.

*4-(2-Isopropyl-5-methylphenoxy)phthalonitrile* (**Pn1**). Yellow solid (8.77 g, 0.032 mol, 79% yield). ^1^H-NMR (CDCl_3_): δ = 7.71 (d, *J* = 8.5 Hz, 1 H; Ar-H), 7.30 (d, *J* = 8.5 Hz, 1 H; Ar-H), 7.22 (s, 1 H; Ar-H), 7.19 (d, *J* = 8 Hz, 1 H; Ar-H), 7.11 (d, *J* = 8 Hz, 1 H; Ar-H), 6.75 (s, 1 H; Ar-H), 2.96 (m, 1 H; C-H), 2.33 (s, 3 H; ArCH_3_), 1.15 (d, *J* = 6.5 Hz, 6 H; CH(CH_3_)_2_). TOF-MS: *m/z* calcd: 299.1 [M+Na]^+^, 315.1 [M+K]^+^; found: 299.1, 314.9. IR (KBr): C-O-C 1242 cm^−1^, CN 2230 cm^−1^. Elemental analysis calcd (%) for C_18_H_16_N_2_O [M = 276.13 g·mol^−1^]: C 78.24, H 5.84, N 10.14; found: C 78.28%, H 5.91%, N 10.02%.

*4-(2-Isopropylphenoxy)phthalonitrile* (**Pn2**). Light yellow solid (8.38 g, 0.032 mol, 79% yield). ^1^H-NMR (CDCl_3_): δ =7.54 (q, *J* = 8.5 Hz, 1 H; Ar-H), 7.42 (t, *J* = 8.5 Hz, 1 H; Ar-H), 7.286 (t, *J* = 8.5 Hz, 2 H; Ar-H), 7.07 (t, *J* = 8 Hz, 1 H; Ar-H), 7.05 (t, *J* = 8 Hz, 2 H; Ar-H), 2.95 (m, *J* = 7 Hz, 1 H; C–H), 1.27 (d, 6H; *J* = 7 Hz, CH(CH_3_)_2_). TOF-MS: *m/z* calcd: 299.1 [M+Na]^+^, 315.1 [M+K]^+^; found: 299.1, 314.9. IR (KBr): C–O–C 1242 cm^−1^, –CN 2230 cm^−1^. Elemental analysis calcd (%) for C_17_H_14_N_2_O [M = 262.1 g·mol^−1^]: C 77.84, H 5.38, N 10.68; found: C 78.80%, H 5.42%, N 10.66%.

*4-(2-Isopropylphenylthio)phthalonitrile* (**Pn3**). Light yellow solid (1.01 g, 0.036 mol, 89% yield). ^1^H-NMR (CDCl_3_): δ = 7.54 (q, *J* = 8.5 Hz, 1 H; Ar-H), 7.42(t, *J* = 8.5 Hz, 1 H; Ar-H), 7.286 (t, *J* = 8.5 Hz, 2 H; Ar-H), 7.07 (t, *J* = 8 Hz, 1 H; Ar-H), 7.05 (t, *J* = 8 Hz, 2 H; Ar-H), 2.95 (m, *J* = 7 Hz, 1 H; C–H), 1.27 (d, *J* =7 Hz, 6 H; CH(CH_3_)_2_). TOF-MS: *m/z* calcd: 301.38 [M+Na]^+^, 315.1 [M+K]^+^; found: 299.1, 314.9. IR (KBr): C–S–C 1240 cm^−1^, –CN 2230 cm^−1^. Elemental analysis calcd (%) for C_17_H_14_N_2_O [M = 262.1 g·mol^−1^]: C 67.75, H 4.68, N 10.64; found: C 67.73%, H 4.68%, N 10.66%.

#### 3.2.2. Synthesis of **1a** and **1b**

NiCl_2_·6H_2_O (95 mg, 0.4 mmol) and **Pn1** (440 mg, 1.6 mmol) were added to 1-octanol (5 mL) and stirred for 0.5 h in succession. After DBU (2 mL) was added, the mixture was sequentially stirred for 21 h and then heated to 160 °C over 8 h under a nitrogen atmosphere. After cooling down, 1-octanol was removed under reduced pressure. The collected solid was purified by column chromatography (silica gel/CHCl_3_–MeOH 20:1) to afford pure **1a** as a green powder in 15% yield (70 mg, 0.05 mmol) and **1b** as a blue powder in 37% yield (172 mg, 0.15 mmol), respectively. 

**1a:** MS(CHCl_3_): found: *m/z* = 1162.9 (M); FT-IR (KBr) cm^−1^: 3080, 3050, 2961, 2924, 2869, 1770, 1735, 1699, 1650, 1613, 1573, 1559, 1535, 1502, 1471, 1412, 1337, 1248 (C–O–C), UV-Vis B band: 299, 332nm; Q band: 609, 675nm. *Anal.* Calcd: C 73.68%; H 5.73 %; N 9.48%; calculated: C 74.29%; H 5.54%; N 9.63%.

**1b**: ^1^H-NMR (500 MHz, CDCl_3_): δ = 7.87 (brs, 12 H; Pc-Ar-H), 7.41 (brs, 4 H; O-Ar-H), 7.10 (brs, 8 H; O-Ar-H), 3.58 (brs, 4 H; tert-C-H), 2.37 (brs, 12 H; Ar-CH3), 1.41 (brs, 24 H; isopropyl-CH_3_). TOF-MS: *m/z* calcd: 1162.4 [M]^+^; found: 1162.9. UV/Vis (CHCl_3_): λ_max_ (logε) = 677 nm (5.35). IR (KBr): C–O–C 1249 cm^−1^. Elemental analysis calcd (%) for C_72_H_64_N_8_O_4_Ni [M = 1162.44 g·mol^−1^]: C 74.29%, H 5.54%, N 9.63%; found: C 74.68%, H 5.73%, N 9.48%. 

The synthesis of Pcs **2a**–**3a** are the same as above; the yields of **2a** and **3a** were 14% and 11%, respectively.

### 3.3. Synthesis of **1a**–**3a** Nanotubes

The **1a**–**3a** molecules are self-organized into nanotubes via a simple solvent diffusion method [36,37]. When methanol was added dropwise into **1a**–**3a** chloroform solution, the **1a**–**3a** molecules are self-assembled into nanotubes. The tubes were then washed carefully by ethanol to remove chloroform.

## 4. Conclusions

In this paper, through H-bonding interaction a series of **1a**–**3a** dimers were constructed by self-assembly as a result of surface accumulation. The dimers are endowed with a novel CT band at around 1d154 nm. Images of **1a** nanotubes are observed by electron microscopy. Our results provide insights into the interaction of molecular dimers and the direct possibility to synthesize materials having potential application in magnets and supramolecular architectures.

## Supplementary Materials

Supplementary materials can be accessed at: http://www.mdpi.com/1420-3049/18/4/4628/s1.
