# Supplementary material for: A Series of Asymmetrical Phthalocyanines: Synthesis and Near Infrared Properties
Source: Molecules. 2013 Apr 19;18(4):4628–39. doi: 10.3390/molecules18044628 (PMC6270185; doi:10.3390/molecules18044628)

Supplementary Materials

Scheme S1. Synthesis of **Pn1-3**.
Scheme S2. The possible mechanism of formation of **1a**.
Figure S1. ^{1}H-NMR of **Pn1-3**.
Figure S2. UV/VIS/NIR of **2a** and **3a**.
Figure S3. ^{1}H-^{13}C-NMR of **2a**.
Figure S4. Hi-Res MALDI of **2a** and MALDI TOF MS of **3a**.
Figure S5. IR of **1a–3a**.
Figure S6. UV-vis/NIR spectrum of **1b**.
Figure S7. The XRD of **2a–3a**.

Scheme S1. Synthesis of **Pn1-3**.

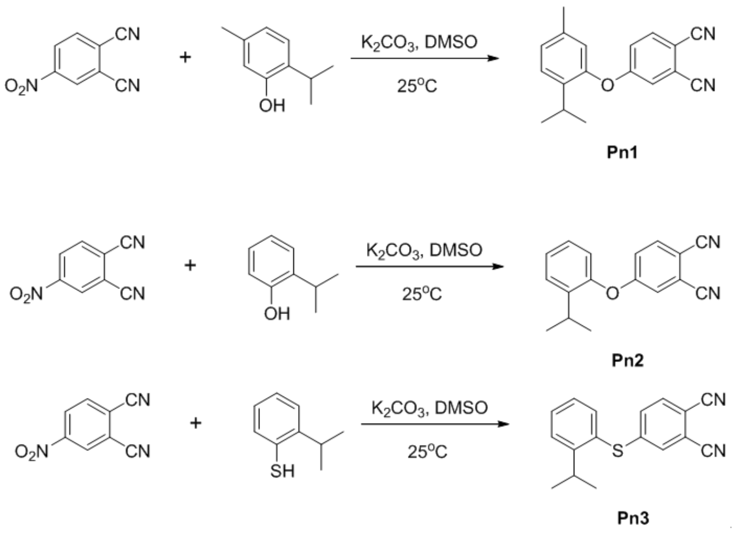

Scheme S2. The possible mechanism of formation of **1a**.

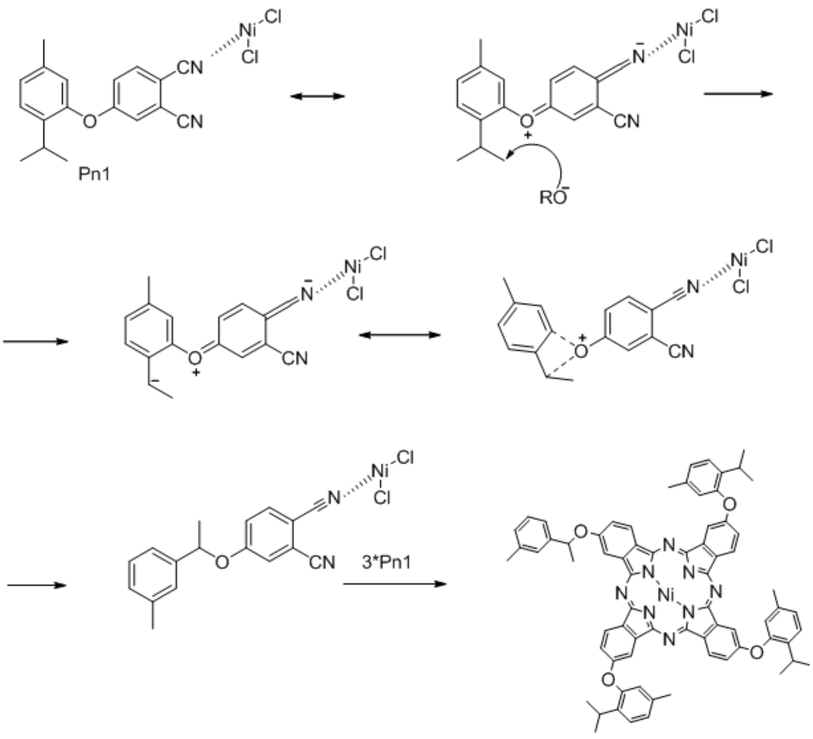

Figure S1. ^{1}H-NMR of **Pn1-3**.

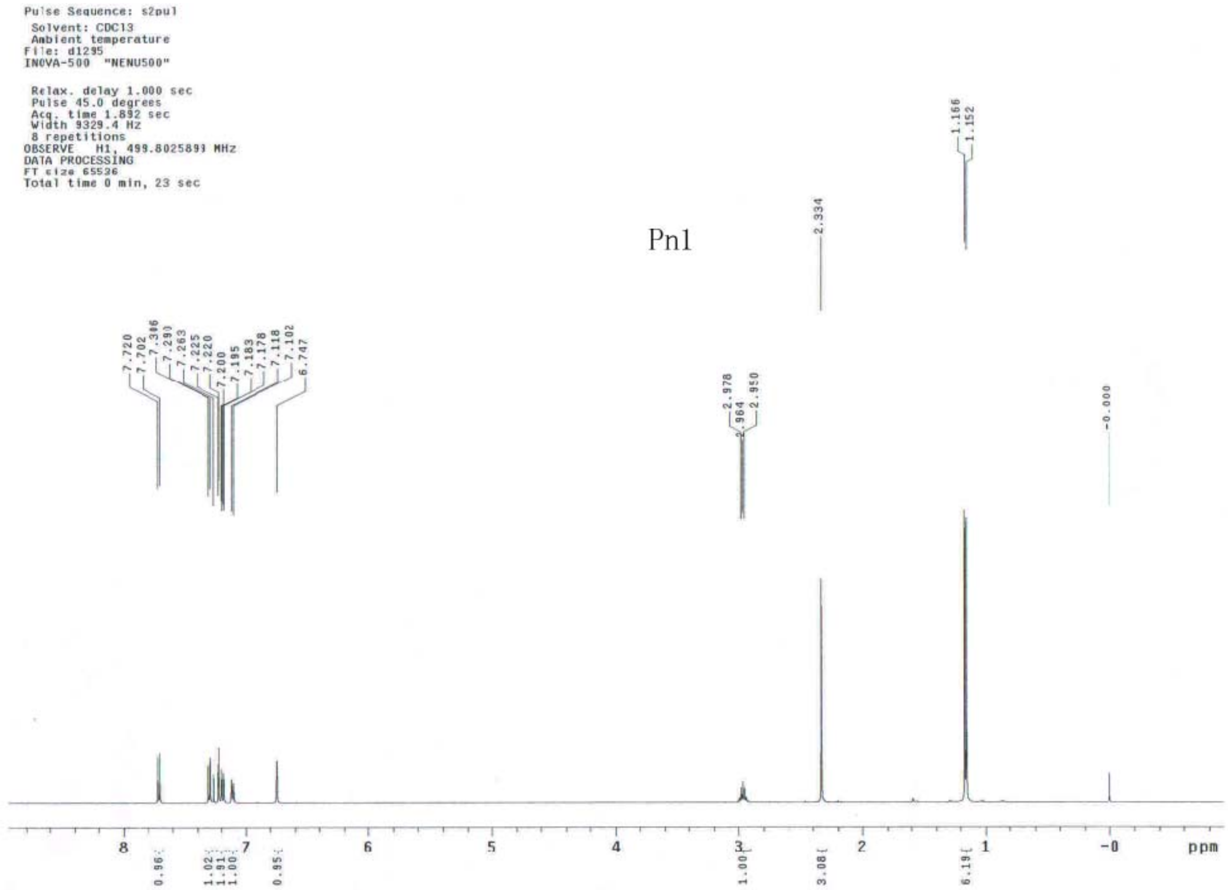

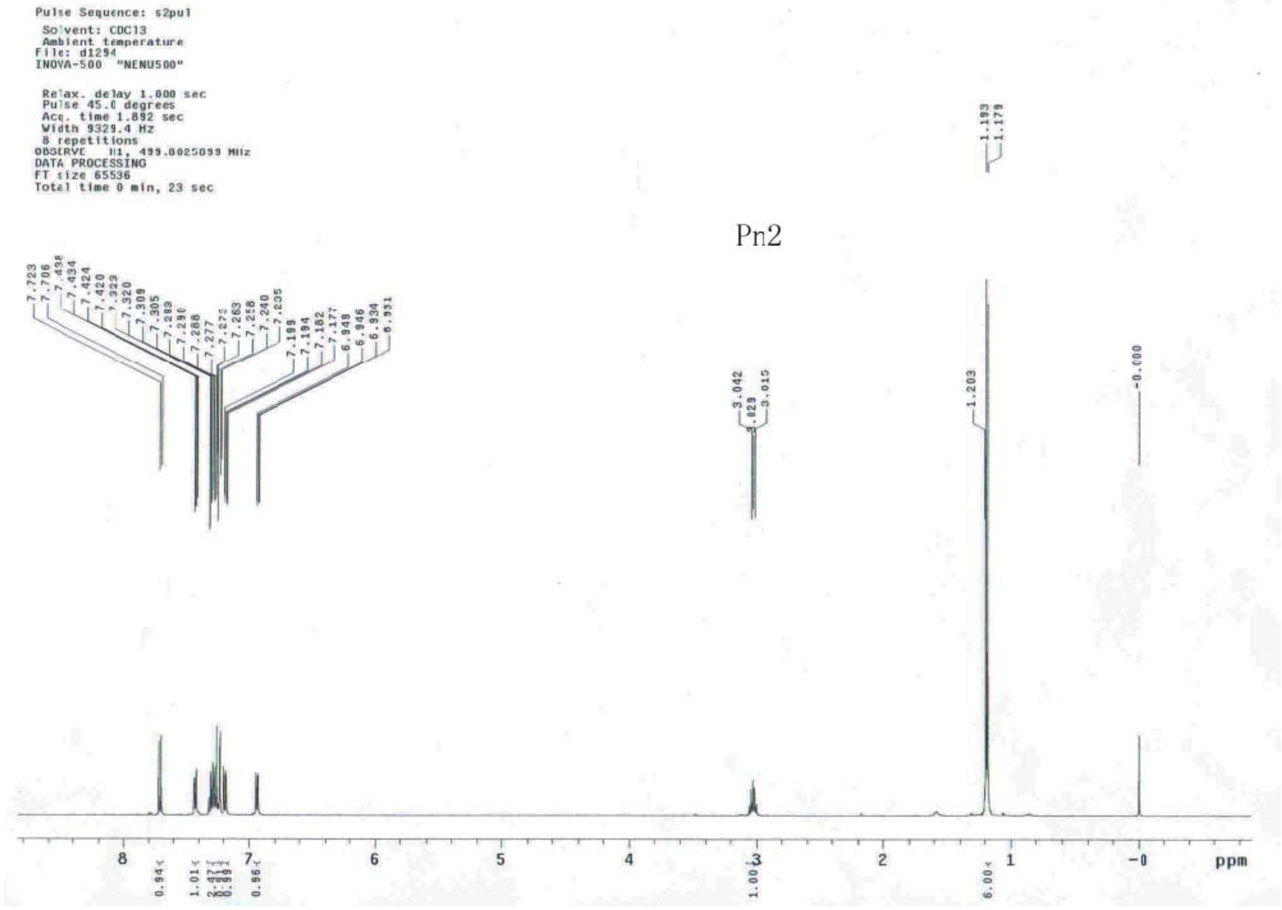

Figure S1. *Cont.*

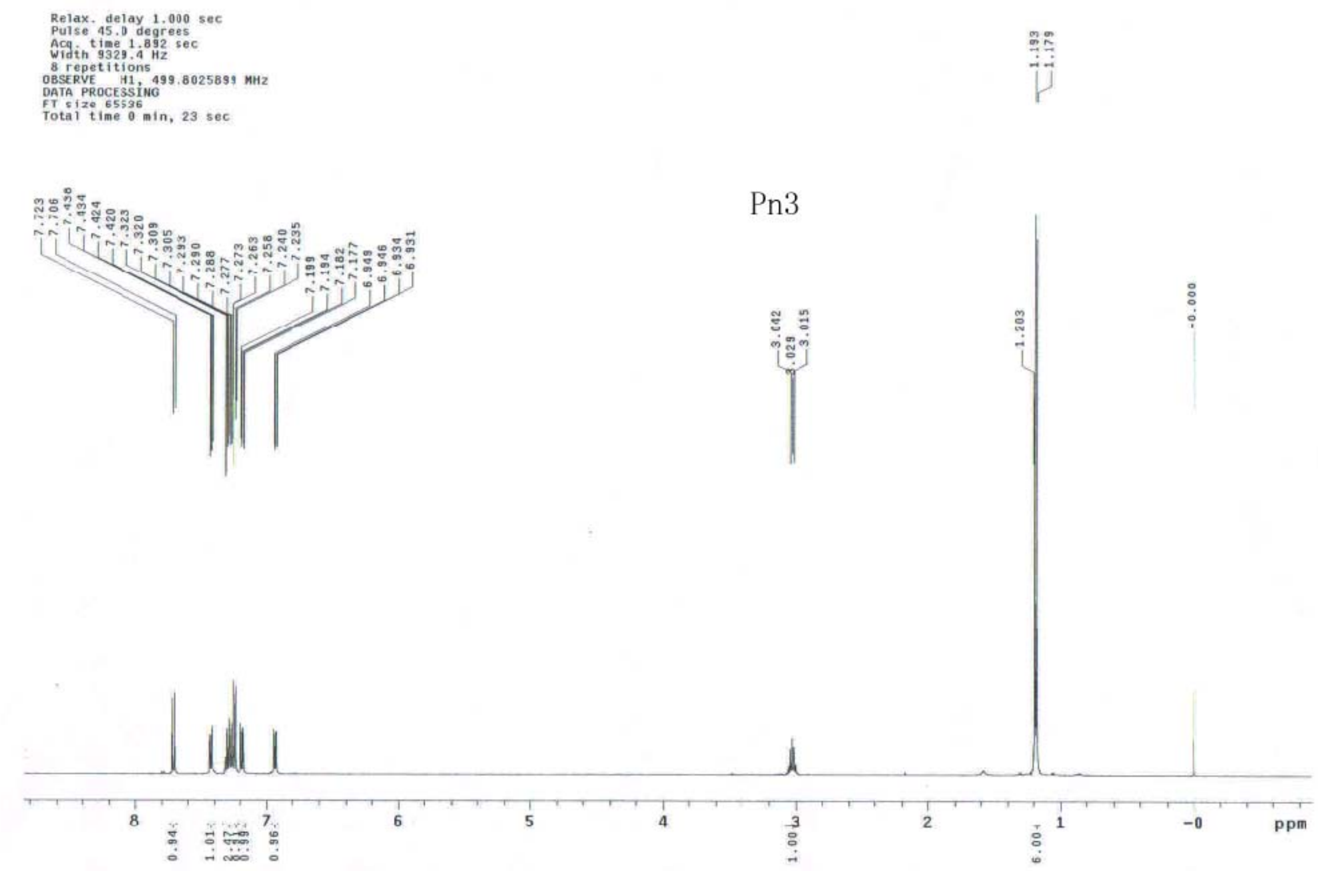

Figure S2. UV/VIS/NIR of **2a** and **3a**.

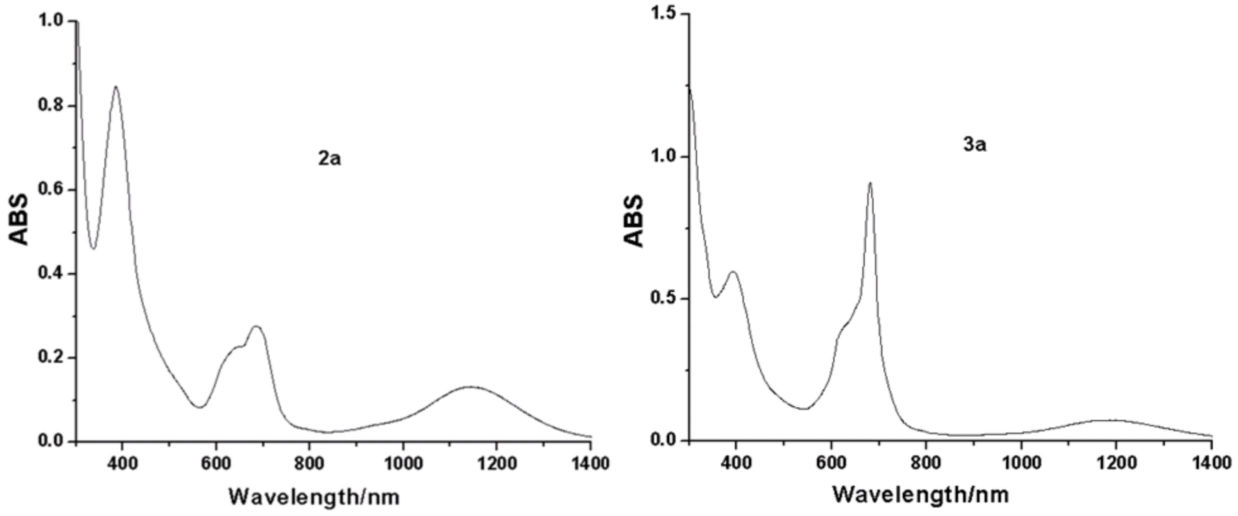

Figure S3. ^1H-^{13}C-NMR of **2a**.

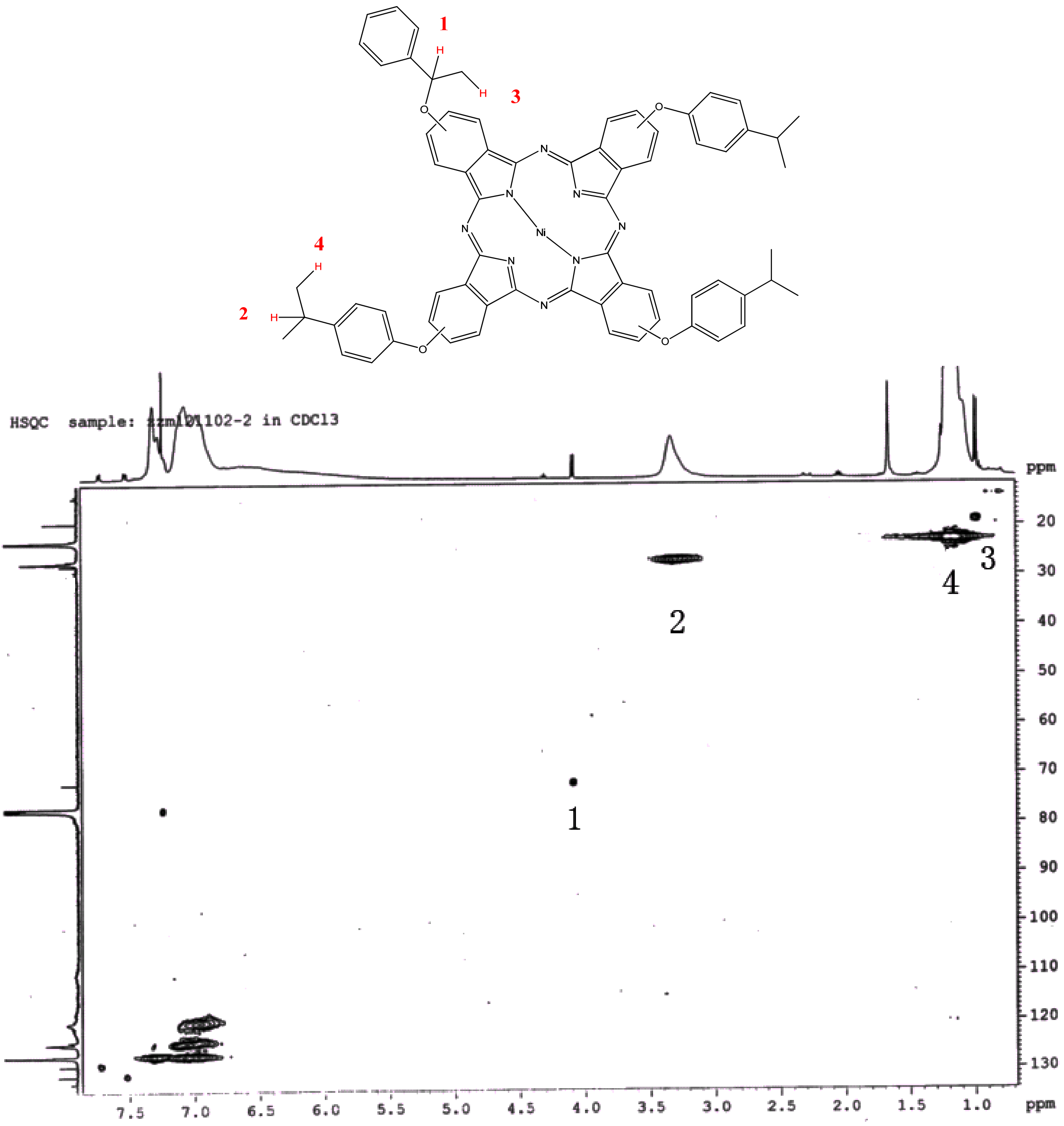

Figure S4. Hi-Res MALDI of **2a** and MALDI TOF MS of **3a**.

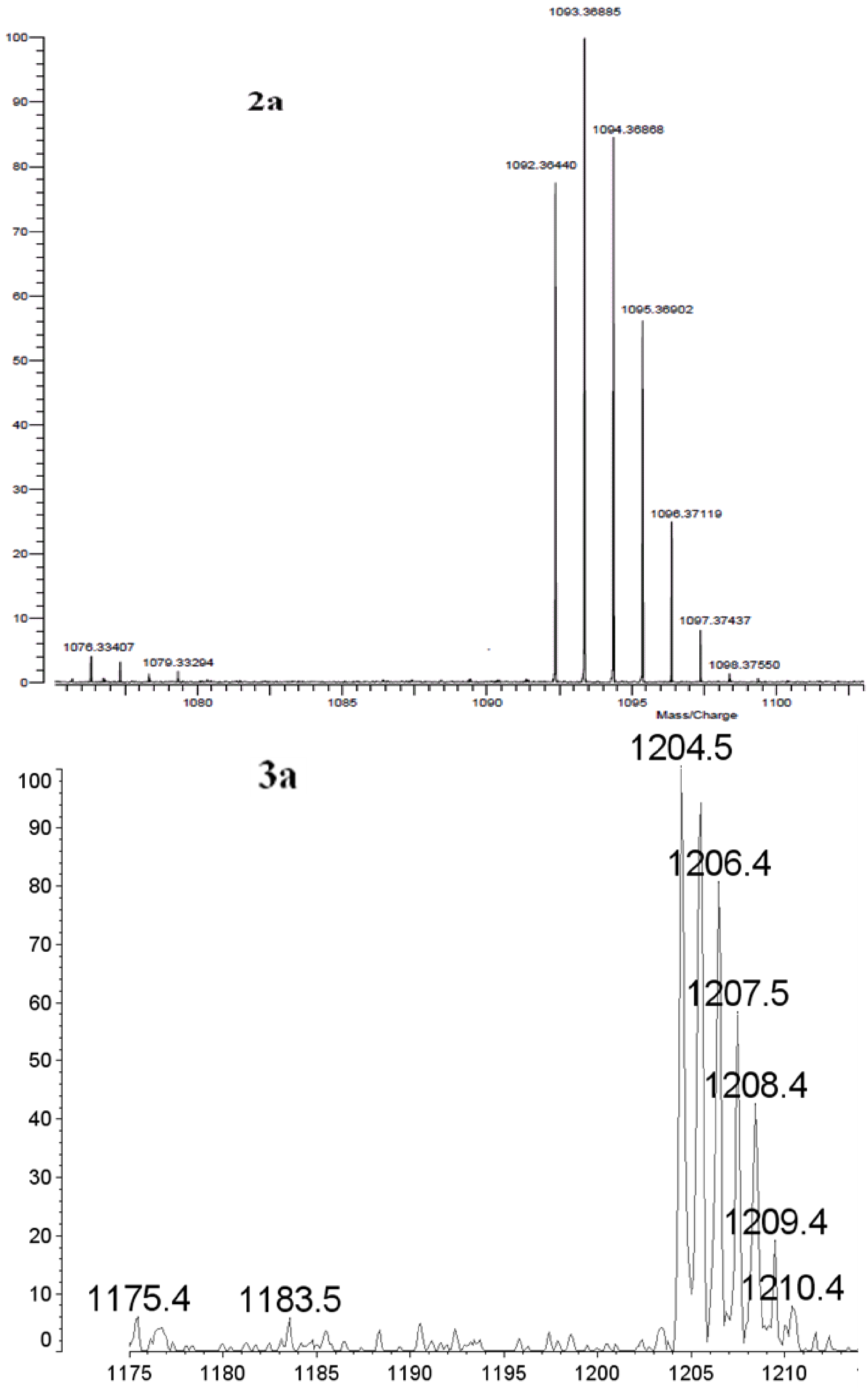

Figure S5. IR of **1a–3a**.

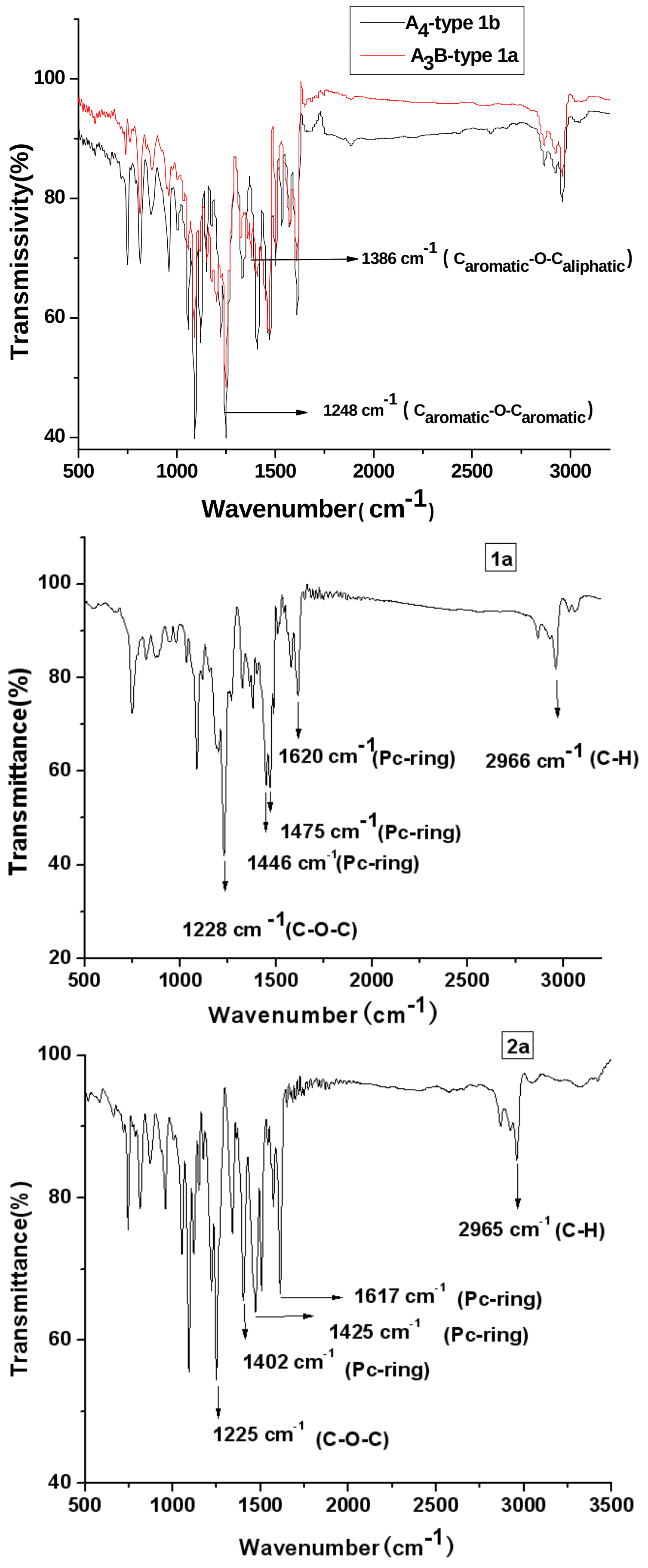

Figure S5. *Cont.*

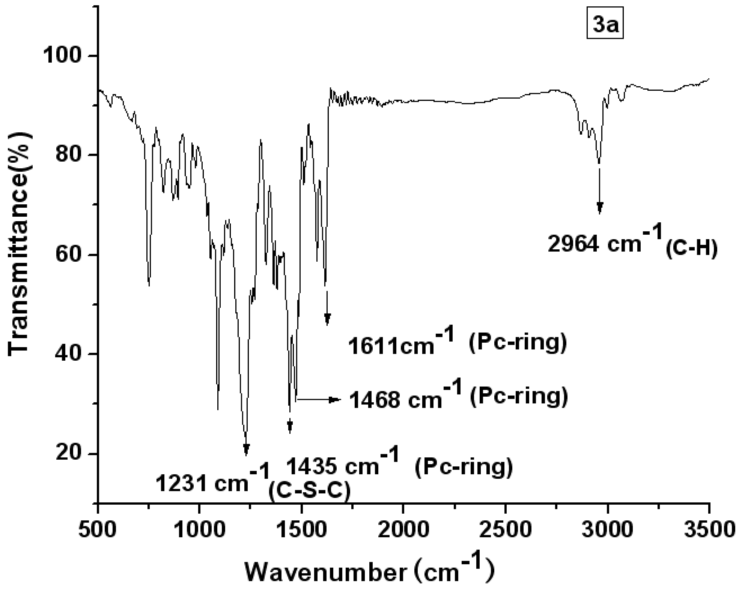

Figure S6. UV-vis/NIR spectrum of **1b**.

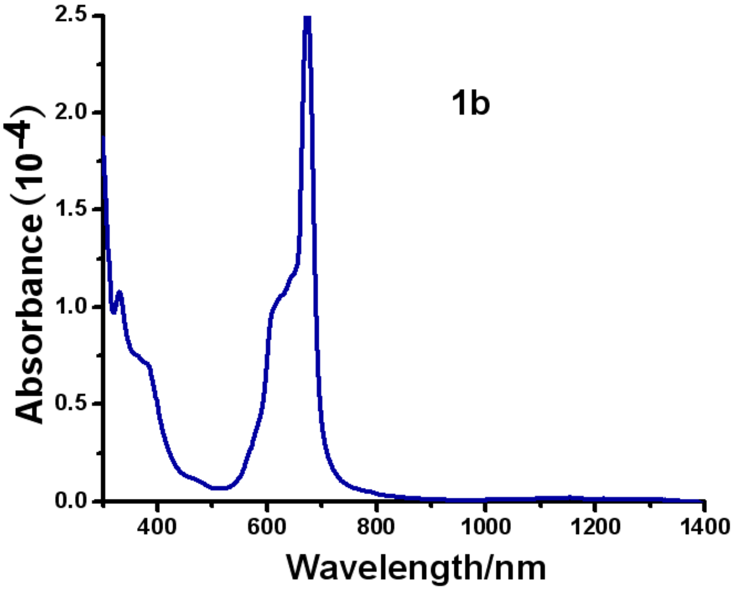

Figure S7. The XRD of **2a**–**3a**.

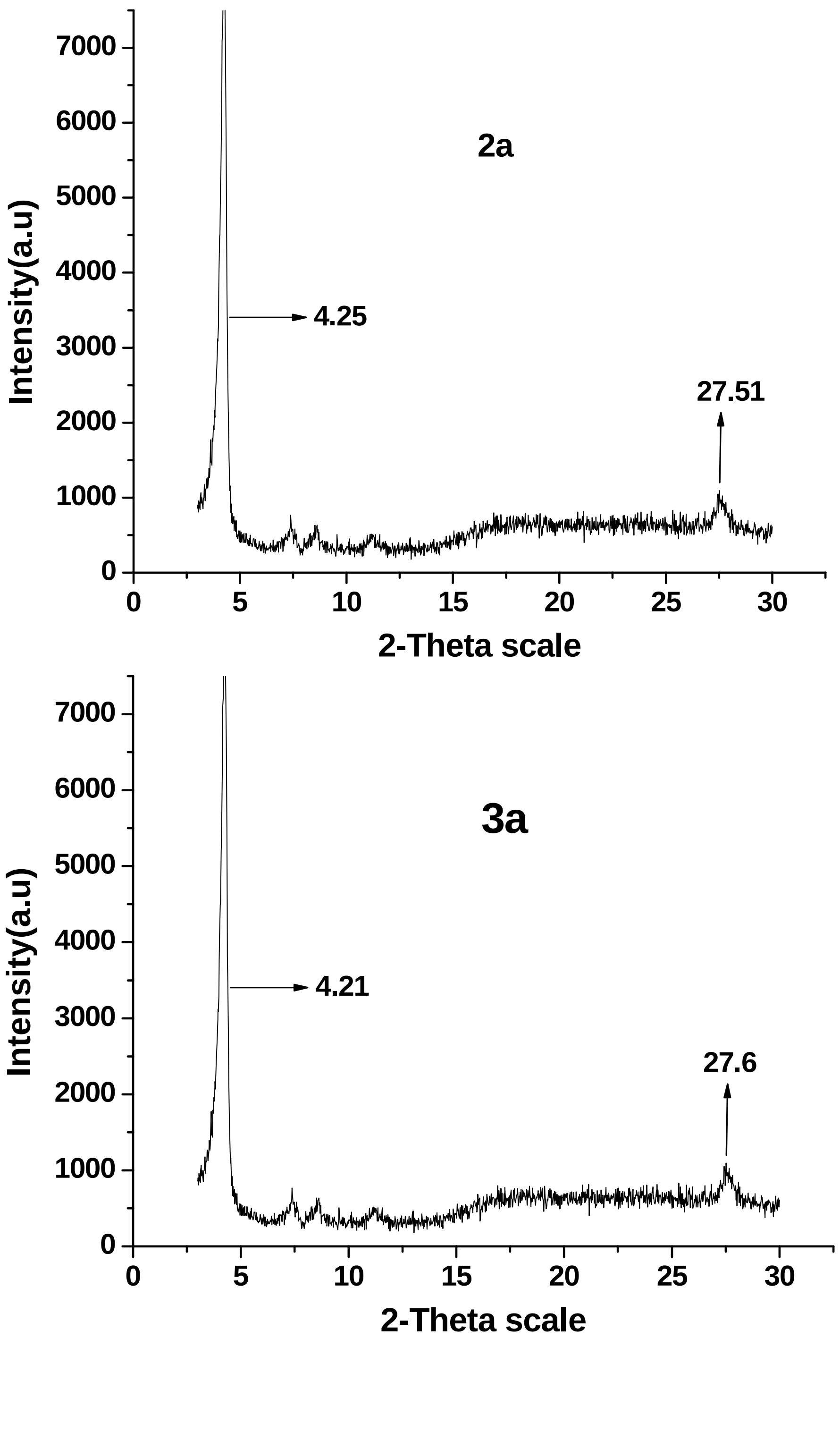

Supplement: Supplementary file 1 [file molecules-18-04628-s001.pdf]
